# The Impact of Pentraxin 3 Serum Levels and Angiotensin-Converting Enzyme Polymorphism on Pulmonary Infiltrates and Mortality in COVID-19 Patients

**DOI:** 10.3390/biomedicines12071618

**Published:** 2024-07-20

**Authors:** Zdravka Krivdić Dupan, Vlatka Periša, Mirjana Suver Stević, Martina Mihalj, Maja Tolušić Levak, Silva Guljaš, Tamer Salha, Domagoj Loinjak, Martina Kos, Matej Šapina, Ivana Canjko, Mirela Šambić Penc, Marin Štefančić, Nenad Nešković

**Affiliations:** 1 Department of Radiology, Osijek University Hospital, 31000 Osijek, Croatia; 2 Medical Faculty Osijek, Josip Juraj Strossmayer University of Osijek, 31000 Osijek, Croatia; 3 Department of Hematology, Osijek University Hospital, 31000 Osijek, Croatia; 4 Department of Transfusion Medicine, Osijek University Hospital, 31000 Osijek, Croatia; 5 Department of Dermatology, Osijek University Hospital, 31000 Osijek, Croatia; 6 Faculty of Dental Medicine, Josip Juraj Strossmayer University of Osijek, 31000 Osijek, Croatia; 7 Department of Internal Medicine, Osijek University Hospital, 31000 Osijek, Croatia; 8 Department of Pediatrics, Osijek University Hospital, 31000 Osijek, Croatia; 9 Department of Radiotherapy and Oncology, Osijek University Hospital, 31000 Osijek, Croatia; 10 Department of Radiology, National Memorial Hospital Vukovar, 32000 Vukovar, Croatia; 11 International Medical Center Priora, 31431 Cepin, Croatia

**Keywords:** pentraxin 3, *ACE* polymorphism, COVID-19, chest X-ray, MBrixia score

## Abstract

Objectives: The aim of this study was to examine the impact of the pentraxin 3 (PTX3) serum level and angiotensin-converting enzyme (ACE) gene insertion/deletion (I/D) polymorphism on the severity of radiographic pulmonary infiltrates and the clinical outcomes of COVID-19. Methods: The severity of COVID-19 pulmonary infiltrates was evaluated within a week of admission by analyzing chest X-rays (CXR) using the modified Brixia (MBrixa) scoring system. The insertion (I)/deletion (D) polymorphism of the *ACE* gene and the serum levels of PTX3 were determined for all patients included in the study. Results: This study included 80 patients. Using a cut-off serum level of PTX3 ≥ 2.765 ng/mL, the ROC analysis (AUC 0.871, 95% CI 0.787–0.954, *p* < 0.001) showed a sensitivity of 85.7% and specificity of 78.8% in predicting severe MBrixa scores. Compared to *ACE* I/I polymorphism, D/D polymorphism significantly increased the risk of severe CXR infiltrates, OR 7.7 (95% CI: 1.9–30.1), and *p* = 0.002. Significant independent predictors of severe CXR infiltrates include hypertension (OR 7.71), PTX3 (OR 1.20), and *ACE* D/D polymorphism (OR 18.72). Hypertension (OR 6.91), PTX3 (OR 1.47), and ACE I/I polymorphism (OR 0.09) are significant predictors of poor outcomes. Conclusion: PTX3 and *ACE* D/D polymorphism are significant predictors of the severity of COVID-19 pneumonia. PTX3 is a significant predictor of death.

## 1. Introduction

The exact pathophysiological mechanism of severe COVID-19 remains unclear. Along with a dysregulated immune response, endothelial dysfunction is emerging as a primary cause of acute respiratory distress syndrome (ARDS) in patients infected with SARS-CoV-2. There is substantial evidence that infection with SARS-CoV-2 leads to endothelial injury with disruption of the endothelial glycocalyx, uncontrolled inflammation, leukocyte adhesion, alterations in endothelial cell permeability, hypercoagulability, and thrombosis [1,2,3]. This immunothrombosis leads to atypical ARDS, characterized by a deterioration in the radiographic appearance of the lungs and worsening hypoxia [4,5]. Angiotensin-converting enzyme 2 (ACE2), expressed in the endothelium and epithelial cells of the respiratory system, is the main entry route for the virus into cells. The binding of SARS-CoV-2 to ACE2 may reduce its availability for regulation of the renin–angiotensin–aldosterone system (RAAS) and promote inflammation by altering the balance between ACE/ACE2 [6,7]. The insertion/deletion (I/D) polymorphism of the *ACE* gene increases ACE activity while decreasing ACE2 activity, contributing to the dysregulation of the ACE/ACE2 balance in the RAAS. Downregulation of ACE2 may be associated with endothelial dysfunction and a higher risk of developing a severe form of COVID-19 [8,9,10]. Also, pentraxin-3 (PTX3), an acute-phase protein, is related to vascular inflammation and endothelial dysfunction by modulating inflammatory cells and decreasing nitric oxide (NO) synthesis within endothelial cells [11]. In this study, we investigated the impact of the *ACE* gene insertion/deletion (I/D) polymorphism and PTX3 levels on the severity of radiographic pulmonary infiltrates and the clinical outcomes of COVID-19.

## 2. Materials and Methods

This study was designed as a cross-sectional study and included patients aged 18 to 83 with PCR-confirmed SARS-CoV-2 infection via nasopharyngeal swab. All patients signed informed consent, and the study was approved by the Ethics Committee of Osijek University Hospital (R1-1510/2023). Patients undergoing active oncological treatment, those vaccinated against SARS-CoV-2, and those with pneumothorax as the dominant finding on radiographs were excluded from the analysis. Data on comorbidities and obesity were obtained from a patient’s medical history. If obesity was not recorded for a patient, it was assumed that the patient was not obese.

### 2.1. COVID-19 and Pulmonary Infiltrates Severity

Patients included in this study were categorized based on the severity of COVID-19 clinical presentation into mild, moderate, and severe/critical illness, in accordance with the National Institutes of Health guidelines [12]. The severity of pulmonary infiltrates was assessed by analyzing antero-posterior or postero-anterior chest X-rays (CXR) using the modified Brixia (MBrixia) scoring system [13]. CXRs selected for analysis were taken either on admission or after clinical deterioration within a week of admission. The MBrixia scoring system divides the CXR into 6 fields for each lung, assessing the intensity of infiltrates with scores ranging from 0 to 3 per field, resulting in a maximum score of 36 points. Patients with mild disease who had no indication for a CXR were not imaged and were assigned a MBrixia score of 0.

### 2.2. ACE Gene Polymorphism and PTX3 Serum Levels

Peripheral blood samples were obtained from all hospitalized patients included in this study within 72 h of CXR imaging. DNA was manually extracted from 200 µL of peripheral blood collected in a vacutainer with K2EDTA using the High Pure PCR Template Preparation Kit (Roche, Indianapolis, IN, USA) according to the manufacturer’s instructions and stored at −20 °C until analysis. Genotyping of *ACE* insertion/deletion (I/D) polymorphisms was performed using a commercial set of specific primers and hybridization probes (LightMix^®^ Kit *ACE* I/D Kit; Tib MolBiol, Berlin, Germany). A 10 µL reaction mix contained MgCl2, Parameter-specific Reagents with specific primers and probes, and Taq polymerase (LightCycler^®^ FastStart DNA Master HybProbe, Roche). After initial denaturation at 95 °C for 10 min, DNA was amplified for 45 cycles with denaturation at 95 °C for 5 s, annealing at 60 °C for 10 s, and extension at 72 °C for 15 s (LightCycler^®^ 480 II, Roche). Genotyping was based on melting curve analysis (Tm) in comparison with the heterozygous (I/D) positive control, which was included in every PCR run. The process of serum separation implied incubation of peripheral blood collected in a vacutainer without anticoagulant for 30 min at room temperature and centrifugation for 10 min at 3000 rpm. Serum aliquots were stored and preserved in a freezer at −80 °C. Serum PTX3 levels were detected by ProcartaPlex™ Immunoassay Kit (Invitrogen, Thermo Fisher Scientific, Waltham, MA, USA) using magnetic bead technology from the Luminex™ and xPonent 4.3 software. A standard curve was generated according to 3-fold serial dilution of the standards. Along with the samples that were analyzed, seven different standard concentrations and blank samples (1X Universal Assay Buffer) were included in the assay. The sample concentrations were calculated by plotting the expected concentrations of the standards against the NET MFI generated by each standard. Data were uploaded to the ProcartaPlex Analysis App (Thermo Fisher Scientific).

### 2.3. Statistical Analysis

Categorical data are presented with absolute and relative frequencies, and numerical data with median and interquartile ranges. The normality of the distribution of numerical data was tested using the Shapiro–Wilk test. Differences in categorical data between groups were tested with the Chi-square test. Differences in continuous variables between two groups were tested using the Mann–Whitney U test, and for three or more groups, the Kruskal–Wallis (with a post hoc Conover test) was used. Receiver operating characteristic (ROC) curve analysis, with a 95% confidence interval (CI), was used to assess the diagnostic accuracy of the MBrixia score and the discriminative ability of PTX3 in distinguishing patients with severe COVID-19 based on the MBrixia score. The optimal discriminatory cut-off values were calculated using the Youden index. Independent predictors of the severity of CXR infiltrates and outcomes were examined using logistic regression analysis. For statistical analysis, MedCalc^®^ Statistical Software version 22.006 (MedCalc Software Ltd., Ostend, Belgium; 2023) and IBM SPSS software (IBM Corp. Released 2021. IBM SPSS Statistics for Macintosh, Version 28.0. Armonk, NY, USA: IBM) were used.

## 3. Results

This study included 80 patients with COVID-19. According to the severity of the clinical presentation, 29 (36%), 22 (28%), and 29 (36%) of them had mild, moderate, and severe/critical illnesses. Demographic characteristics, comorbidities, CXR MBrixia score, ACE gene polymorphism, and disease outcomes regarding the severity of the clinical presentation are shown in Table 1.

Only one patient in the mild group required mechanical ventilation, while among critically ill patients, 21 (72.4%) were mechanically ventilated. The MBrixa score differed significantly among the different levels of COVID-19 severity (Table 1). With an AUC of 0.998 (0.994–1.000), *p* < 0.001, MBrixia cut-off value ≥ 23.5 has a specificity of 96.6% and a sensitivity of 100% in diagnosing the critical form of COVID-19 (AUC 0.998 (0.994–1.000), *p* < 0.001) and predict mechanical ventilation with specificity of 86.2% and a sensitivity of 90.9% (AUC 0.897 (0.824–0.971), *p* < 0.001).

**Figure 1:**
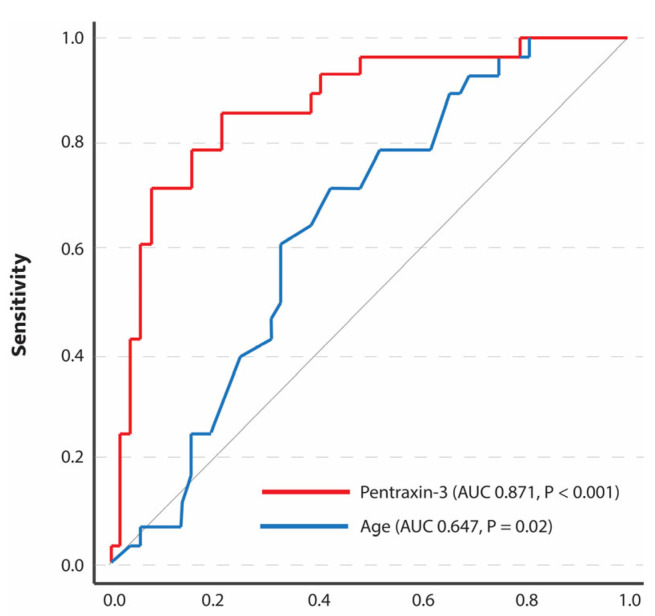
ROC analysis of pentraxin-3 and age in predicting CXR infiltrate severity.

PTX3 serum levels were significantly higher in the group of patients with MBrixia higher than 23.5 compared to those with a lower MBrixia score: 5.45 (3.19–11.78) ng/mL vs. 1.79 (1.14–2.66) ng/mL, respectively, *p* < 0.001. In our sample of patients, PTX3 had better accuracy than age in the prediction of the severity of pulmonary infiltrates, with an AUC of 0.871 (0.787–0.954), *p* < 0.001, showing a sensitivity of 85.7% and a specificity of 78.8% at cut-off values ≥ 2.76 ng/mL in the prediction of a high MBrixa score (Figure 1 and Table 2).

Considering the significant difference in the distribution of *ACE* gene polymorphisms among different severities of COVID-19 (Table 1), the odds ratios (OR) for developing more severe CXR infiltrates, using I/I as the reference point, are shown in Table 3.

Univariate regression analysis showed that hypertension (OR 15.2), diabetes (OR 7.05), and obesity (OR 4.14) are the most significant predictors of CXR infiltrate severity, together with *ACE* D/D polymorphism (OR 3.58) and PTX3 serum level (OR 1.26). In the multivariate regression analysis, only hypertension, PTX3, and *ACE* D/D polymorphism remained important predictors of the severity of CXR infiltrates. The regression model is significant (χ^2^ (6) = 46.707, *p* < 0.001) and correctly classifies 85% of cases (Table 4).

The most significant predictors of death in the regression analysis were hypertension (OR 14.0), diabetes (OR 3.87), PTX3 (OR 1.57), and *ACE* D/D polymorphism (OR 2.96). In the multivariate regression model, important predictors of death were hypertension and PTX3, while *ACE* I/I polymorphism reduced the probability of death (Table 5). The model is statistically significant (χ^2^ (3) = 46.629, *p* < 0.001) and correctly classifies 85% of cases.

## 4. Discussion

The results of this study showed that serum PTX3 levels and *ACE* I/D polymorphism are important predictors of severity of COVID-19 lung infiltrates. SARS-CoV-2 infection can present in a wide clinical spectrum, from asymptomatic cases to severe illness with acute hypoxic respiratory failure and ARDS. Severe respiratory complication often leads to multiorgan failure, which is the main cause of death from COVID-19 [14,15,16]. Lung damage caused by SARS-CoV-2 is highly heterogeneous and includes virus-induced pulmonary hemorrhage, immune cell alveolar infiltration, and thrombosis, along with disruption of the alveolar wall and fibroblast proliferation [17]. Pulmonary microthrombosis and endothelial injury are extensive in ARDS caused by SARS-CoV-2, unlike ARDS caused by other respiratory viruses [18,19]. Postmortem records indicate that alveolar microthrombi are present in more than 70% of patients who died from COVID-19 [20]. Because of this, endothelial injury accompanied by immunothrombosis emerged as a primary cause of SARS-CoV-2-induced ARDS [21,22]. The role of the endothelium in the pathogenesis of severe COVID-19 has been extensively studied. Endothelial integrity and intact glycocalix are important in maintaining the capillary–alveolar barrier. Endothelial disruption leads to a pro-inflammatory and pro-thrombotic state by causing hyperpermeability of the alveolar-capillary membrane, upregulation of adhesion molecules, leukocyte infiltration, platelet aggregation, and activation of the coagulation system, resulting in the formation of microthrombi [2,23]. PTX3 is an acute-phase inflammatory glycoprotein and a marker of endothelial damage that is released from endothelial cells and macrophages after stimulation by inflammatory cytokines. It reduces the synthesis of nitric oxide, inhibits endothelial cell proliferation, and promotes vascular inflammation and endothelial dysfunction [11,24,25]. A meta-analysis by Capra et al., which included 12 clinical studies evaluating PTX3 plasma levels in COVID-19 patients, demonstrated that high PTX3 levels are indicative of severe COVID-19 and correlate with poor outcomes [26]. Additionally, its potential role in endothelial damage was demonstrated in the study by Lapadula et al., which showed that PTX3 plasma levels are significantly higher in COVID-19 patients with thrombotic complications [27]. Our results showed that the serum level of PTX3 correlates with the MBrixa score and is three times higher in patients with an MBrixa score above 23.5 compared to those with less severe infiltrates. This supports the hypothesis that pulmonary infiltrates in COVID-19 may be caused by endothelial injury and microthrombosis. Additionally, the PTX3 serum level serves as a reliable predictor of the severity of CXR infiltrates and poor outcomes, consistent with previous studies [27,28,29].

Direct damage to the pulmonary endothelium by SARS-CoV-2 is mediated by the membrane-bound ACE2 receptor expressed on endothelial cells, through which the virus enters the cell. ACE2 is a homolog of ACE and is an important component of the RAAS system. While ACE catalyzes the production of angiotensin II, which has vasoconstrictive and inflammatory properties, ACE2 converts angiotensin II into angiotensin 1-7, which has vasodilatory and anti-inflammatory properties. ACE/ACE2 balance plays an important role in the regulation of blood pressure, vascular tone, and inflammation [6]. Downregulation of ACE2 as a therapeutic target could potentially reduce viral entry into cells. However, decreased ACE2 activity would also diminish the production of angiotensin 1-7, which possesses anti-inflammatory properties, and could exacerbate the clinical condition of already infected patients [30,31]. Various risk factors such as age, male gender, and comorbidities including diabetes, arterial hypertension, cardiovascular diseases, and obesity have an impact on COVID-19 severity [32,33,34]. The results of our study align with previous research, showing that comorbidities such as hypertension, diabetes, and obesity are significant independent predictors of the CXR infiltrates severity. Interestingly, many of these comorbidities are closely related to *ACE* I/D polymorphisms [35,36,37,38,39,40,41]. Additionally, studies have shown that the *ACE* I/D polymorphism plays an important role in coagulation and that the *ACE* D allele is associated with higher levels of plasminogen activator inhibitor (PAI-1), particularly in men and postmenopausal women, increasing the risk of thrombotic events [42,43,44,45,46]. Given the role of these polymorphisms in the ACE/ACE2 balance, this may represent a genetic risk factor for severe COVID-19 infection and CXR infiltrates. Previous studies have shown a strong relationship between the D allele of the *ACE* I/D polymorphism, clinical severity, and outcome of COVID-19 [10,47]. The D/D genotype is associated with the highest level of ACE [48], therefore promoting a pro-inflammatory state during COVID-19. In our sample of patients, the presence of the D allele is a strong independent predictor of CXR infiltrate severity, increasing the risk of severe pulmonary infiltrates in homozygotes by nearly nineteen times. A shortcoming of this research is the limited sample size and its observational nature, which make it challenging to draw definitive conclusions about the underlying mechanisms of CXR infiltrate pathogenesis. Also, comorbidities such as chronic obstructive pulmonary disease and heart, liver, or kidney disease can significantly impact COVID-19 severity, but they were not analyzed in this study. Further large-scale, prospective analyses are needed to validate our results and explore COVID-19 pathogenesis in greater detail.

## 5. Conclusions

The results of this study showed that the serum levels of PTX3 and *ACE* I/D polymorphism play a significant role in COVID-19 CXR severity. The strong correlation between PTX3 serum levels, *ACE* I/D polymorphism, and the MBrixia score supports the hypothesis that endothelial injury and genetic factors are important in the pathogenesis of COVID-19.

## Figures and Tables

**Table 1:** Patient’s characteristics and outcome regarding COVID-19 severity.

	COVID-19 Severity [n (%) or Median (IQR)]			p *
	Mild (n = 29)	Moderate (n = 22)	Severe/Critical (n = 29)	
Age (years)	54 (35–64)	68 (62–79)	70 (62–78)	<0.001
Sex (male)	10 (34.5)	10 (45.5)	18 (62.1)	0.10
Comorbidity				
Dyslipidemia	3 (10.3)	6 (27.3)	10 (34.5)	0.09
Obesity	3 (10.3)	6 (27.3)	13 (44.8)	0.01
Diabetes	2 (6.9)	3 (13.6)	12 (41.4)	0.003
Hypertension	8 (27.6)	15 (68.2)	27 (93.1)	<0.001
MBrixia score	0 (0–1)	16.5 (14–18.5)	30 (26–32.5)	<0.001
*ACE* polymorphism				
I/I	15 (51.7)	7 (31.8)	5 (17.2)	0.02
I/D	11 (37.9)	9 (40.9)	12 (41.4)	0.96
D/D	3 (10.3)	6 (27.3)	12 (41.4)	0.03
Outcome (died)	1 (3.4)	3 (13.6)	23 (79.3)	<0.001

* Chi-square test for categorical and Kruskal–Wallis test for continuous variable; ACE: angiotensin-converting enzyme gene.

**Table 2:** The ROC curve parameters for PTX3 and age in predicting CXR severity.

	AUC (95% CI)	Sensitivity	Specificity	Cut-Off	Youden	*p*
Age	0.647 (0.526–0.767)	71.4%	57.7%	≥65.5 y	0.291	0.02
PTX3	0.871 (0.787–0.954)	85.7%	78.8%	≥2.76 ng/mL	0.645	<0.001

AUC—area under the curve; CI—confidence interval; PTX3—pentraxin-3.

**Table 3:** Odds ratio for severe CXR infiltrate with regards to ACE gene polymorphism.

		MBrixia Score [n (%)]		OR (95% CI)	p *
		Score < 23.5 (n = 52)	Score ≥ 23.5 (n = 28)		
*ACE* polymorphism	I/I	23 (44.2)	4 (14.2)	1	
	I/D	20 (38.5)	12 (42.9)	3.4 (0.9–12.4)	0.05
	D/D	9 (17.3)	12 (42.9)	7.7 (1.9–30.1)	0.002

* Chi-square test; OR: odds ratio; CI: confidence interval; *ACE*: angiotensin-converting enzyme gene.

**Table 4:** Logistic regression assessing the probability of severe CXR infiltrates.

	ß	Wald	*p*	OR	95% CI
Univariate regression					
Sex (F)	−0.83	2.96	0.08	0.44	0.17–1.12
Age	0.04	4.97	0.03	1.04	1.00–1.08
Dyslipidemia	0.97	3.29	0.07	2.65	0.92–7.62
Obesity	1.42	7.26	0.007	4.14	1.47–11.63
Diabetes	1.95	10.39	0.001	7.05	2.15–23.12
Hypertension	2.72	12.01	<0.001	15.2	3.26–70.62
PTX3	0.23	8.61	0.003	1.26	1.08–1.46
*ACE* D/D polymorphism	1.28	5.81	0.02	3.58	1.27–10.11
*ACE* I/D polymorphism	0.18	0.15	0.70	1.20	0.47–3.05
*ACE* I/I polymorphism	−1.56	6.58	0.01	0.21	0.06–0.69
Multivariate regression					
Obesity	1.31	2.99	0.08	3.69	0.84–16.21
Diabetes	1.51	3.20	0.07	4.52	0.86–23.64
Hypertension	2.01	4.62	0.03	7.71	1.20–50.16
PTX3	0.18	6.19	0.01	1.20	1.04–1.38
*ACE* D/D polymorphism	2.93	7.32	0.007	18.72	2.24–156.20
*ACE* I/D polymorphism	1.65	2.61	0.11	5.19	0.70–38.30
Constant	−5.56	15.52	<0.001		

OR: odds ratio; CI: confidence interval; PTX3: pentraxin 3; *ACE*: angiotensin-converting enzyme gene.

**Table 5:** Logistic regression assessing the probability of death outcome.

	ß	Wald	*p*	OR	95% CI
Univariate regression					
Sex (F)	−1.19	5.77	0.02	0.30	0.11–0.80
Age	0.06	8.48	0.004	1.06	1.02–1.10
Dyslipidemia	0.18	0.11	0.74	1.19	0.41–3.05
Obesity	0.69	1.83	0.18	2.01	0.73–5.53
Diabetes	1.35	5.65	0.02	3.87	1.27–11.78
Hypertension	2.64	11.31	<0.001	14.0	3.01–65.17
PTX3	0.45	12.90	<0.001	1.57	1.23–2.00
*ACE* D/D polymorphism	1.08	4.25	0.04	2.96	1.05–8.28
*ACE* I/D polymorphism	0.28	0.34	0.56	1.32	0.51–3.38
*ACE* I/I polymorphism	−1.48	5.94	0.01	0.23	0.07–0.75
Multivariate regression					
Hypertension	1.93	4.37	0.04	6.91	1.13–42.37
PTX3	0.38	9.98	0.002	1.47	1.17–1.86
*ACE* I/I polymorphism	−2.37	4.84	0.03	0.09	0.01–0.77
Constant	−3.28	12.31	<0.001		

OR: odds ratio; CI: confidence interval; PTX3: pentraxin 3; *ACE*: angiotensin-converting enzyme gene.

## Data Availability

The data presented in this study are available on request from the corresponding author.
